# Right coronary artery stenting as treatment of postoperative bleeding after cardiac surgery

**DOI:** 10.1186/1749-8090-8-S1-P85

**Published:** 2013-09-11

**Authors:** J Rubio Alvarez, J Sierra Quiroga, B Adrio Nazar, JM Martinez Cereijo, D Otero, L Reija Lopez, A Granda Bauza, C Rubio Taboada

**Affiliations:** 1 Cardiac Surgery, University Hospital, Santiago de Compostela, Spain; 2 Cardiology, University Hospital, Santiago de Compostela, Spain; 3 Vascular Surgery, University Hospital, Elche, Spain

## 

Angiosarcomas are the most common primary malignant neoplasms of the heart . Coronary perforation is a rare but serious complication of percutaneous coronary intervention with important bleeding into de pericardium, however, this complication can be tackle successfully by covered stents. A 50-year-old man visited the emergency service because of palpitations and left chest pain. A transthoracic echocardiogram was performed and detected a right atrial mass that infiltrated the right atrial free wall and that protruded into the right atrial . For further evaluation of this mass, magnetic resonance imaging and computed tomography were performed . These explorations showed a large excentric tumor in the right atrial free wall, protruding into the right atrial . The tumor extended into the right atrioventricular groove. Coronary angiography showed a right coronary artery with colateral circulation to a big mass. The surgery was performed under standard extracorporeal circulation . The right atrial was excised and the tumour could only be partially resected because it extended right ventricle free wall and tricuspid valve annulus. The right atrial was reconstructed using bovine pericardium and after declamp a persistent bleeding was observed. Because the bleeding control was no possible, we decided to close the chest and to perform a right coronary angiography which had revealed an important free extravasation of contrast into the pericardium throught the colateral circulation . These branches were tackled successfully by covered stents and post-covered stent angiogram showed complete cessation of contrast extravasation . The postoperative course was uneventful and after asymptom-free survival of twenty-three months the patient presented with bone metastases .

